# Musculoskeletal Disorders and Diseases: Biomechanical Modeling in Sport, Health, Rehabilitation and Ergonomics

**DOI:** 10.3390/bioengineering12030300

**Published:** 2025-03-16

**Authors:** Philippe Gorce

**Affiliations:** 1 International Institute of Biomechanics and Occupational Ergonomics, 83418 Hyères, France; gorce@univ-tln.fr; 2 University of Toulon, CS60584, 83041 Toulon, France

Protecting people at work and at leisure, and improving their quality of life, is one of the major challenges faced in this century. From this perspective, understanding the mechanisms that lead to the development of musculoskeletal disorders and diseases is a major multidisciplinary scientific challenge. This Special Issue is dedicated to recent advances in biomechanical modeling research used to explore and understand the musculoskeletal system (macro- and microscopic). Computational techniques, biomechanical computation tools and numerical tools enable us to quantify and qualify the most important parameters (biomechanical, physiological, biological or environmental) involved in the onset, prevention and reduction in the effects of musculoskeletal disorders and/or the development of musculoskeletal diseases. They can be used as a complement to experimental protocols, clinical studies, process design, ergonomics, etc., to study, evaluate and understand various situations in life, such as repeated movements in the workplace, evaluation of leisure-time physical activities, analysis of sporting gestures to assess performance, the design of new equipment to compensate for a motor impairment, the proposal of new recommendations in a clinical setting, etc. We support all articles promoting the latest research in the fields of sport, health, rehabilitation and ergonomics that contribute to improving people’s health and quality of life.

The Special Issue “Musculoskeletal disorders and diseases: biomechanical modeling in sport, health, rehabilitation and ergonomics” brings together ten high-quality publications focusing on new advances and applications in the prevention and understanding of musculoskeletal disorders and diseases.

In this context, the use of biomechanical gait models represents an original approach. Xiang et al. utilized a two-degree-of-freedom inverted pendulum gait model with a roll factor to identify different gait styles [1]. Goo et al. exploited a “virtual controller” for pediatric gait rehabilitation. In their work, the authors experimentally compare this virtual controller with a conventional position-tracking controller associated with a questionnaire [2]. The study of ergonomic risks and postures is also helping to expand knowledge of the risk factors and prevalence of musculoskeletal disorders. Han et al. propose a “skeletal compensation” method using convolutional networks of enhanced spatio-temporal graphs to assess MSD risk in healthcare workers. The proposed method is compared with other postural assessment methods and qualified using the REBA score [3]. The study of MSD risks in sport is also the subject of more recent research. For example, Gorce et al. studied the risks of MSD during the tennis serve to protect athletes while maintaining performance. Using the ergonomic Rapid Entire Body Assessment (REBA) tool at each time step and a 3D kinematic analysis of joint angles, the authors assessed the impact of slow and fast serves on the risk of MSD [4]. Wang et al. investigate the biomechanical impact of the split-step technique on forehand and backhand lunges in badminton on injury risk. The results highlight the potential of split-stepping (reduced ground reaction forces, reduced knee flexion on ground contact, etc.) in swing techniques, performance improvement and injury reduction [5]. The use of technical or therapeutic means makes it possible to propose solutions and recommendations to reduce injuries or pathologies of the musculoskeletal system. The application of dynamic taping to prevent the risk of cruciate ligament injury in fatigued soccer athletes has been studied by Wu et al. Dynamic taping, particularly using the spiral technique, appears to attenuate defective landing biomechanics and offer protective benefits [6]. Studying the impact of gait asymmetry on the stability and coordination of dynamic movements also contributes to a better understanding of the mechanisms that lead to the onset of MSD. Liu et al. have shown that early identification of loading patterns enables the development of targeted interventions to prevent foot pathology in children [7]. Fall prevention is also a field that contributes to better identification and diagnosis of musculoskeletal diseases and disorders. Hernandez-Laredo et al. have proposed a fall risk classification method using a Bayesian approach and the simulated annealing algorithm [8]. Analysis of the flexion–relaxation phenomenon of back muscles is important in the development of musculoskeletal disorders. In this context, Chen et al. have explored the influence of leg posture control on the activity of certain muscles, along with the value of such information in the design of prevention protocols [9]. Finally, animal studies have been found to contribute to a better understanding of the causes of bone loss, which increases the risk of fractures and morbidity. Weiser et al. reported on the effects of a combined treatment strategy (neurotrophin transplantation and bodyweight treadmill training) on bone loss in animals with T9–T19 spinal cord injury [10].

In summary, the publications in this Special Issue mark a significant step forward in the field of musculoskeletal disorders and diseases in the workplace and during leisure time, with the aim of improving people’s quality of life. We would like to express our sincere gratitude to all the authors and reviewers who contributed to this Special Issue, and to the *Bioengineering* magazine team for their invaluable help and support.
